# Heptanol decreases the incidence of ischemia-induced ventricular arrhythmias through altering electrophysiological properties and connexin 43 in rat hearts

**DOI:** 10.3892/br.2014.247

**Published:** 2014-03-12

**Authors:** BING SUN, XIANGQIAN QI, JINFA JIANG

**Affiliations:** 1 Department of Cardiology, Tongji Hospital, Tongji University, Shanghai, P.R. China; 2 Department of Cardiology, Taida Cardiology Hospital, Tianjin, P.R. China

**Keywords:** myocardial ischemia, heptanol, ventricular arrhythmia, gap junction, electrophysiology

## Abstract

Heptanol is a type of gap junction inhibitor that decreases electrical conduction velocity. However, little is known regarding the effects of heptanol on the arrhythmias induced by regional myocardial ischemia. This study aimed to investigate the effects of heptanol on ventricular arrhythmias and the underlying mechanisms. On the Langendorff apparatus, isolated hearts of Sprague-Dawley rats underwent 30 min of ischemia, with or without pretreatment with heptanol (0.1, 0.3 or 0.5 mM), 15 min prior to the induction of regional ischemia through ligation of the left anterior descending coronary artery. The incidence of ventricular tachycardia (VT) and ventricular fibrillation (VF) were recorded after ligation. Heptanol decreased the incidence of ventricular arrhythmias (45% in the control group vs. 10% in the 0.1 mM group, 0% in the 0.3 mM group and 0% in the 0.5 mM group, P<0.05), whereas it prolonged the PR interval, QT interval and monophasic action potential duration at 90% repolarization (MAPD90). As evaluated with immunofluorescence microscopy, heptanol was able to partly reverse the downregulation of connexin 43 (Cx43) induced by ischemia. The results of the reverse transcription-polymerase chain reaction were consistent with those of immunofluorescence. In conclusion, heptanol significantly decreased the incidence of VT and VF induced by regional ischemia and prolonged the PR interval, QT interval and MAPD90. Heptanol also partly reversed the downregulation of Cx43 induced by ischemia.

## Introduction

Gap junctions are special regions adjoining cell membranes and are direct pathways for the exchange of signals among cardiac myocytes. Gap junctions mediate current flow, thereby coordinating the spread of excitation and subsequent contraction throughout the myocardium (1). The electrical conduction velocity in the region of gap junctions is faster compared to that elsewhere (2). It was previously suggested that changes in the gap junctions in morbid heart tissue, collectively referred to as gap junction remodeling, appear to be associated with the incidence of arrhythmias (3–7).

Heptanol is commonly used as a gap junction inhibitor in several experiments. Previous studies demonstrated that regional perfusion with heptanol may decrease the conduction velocity and induce reentrant arrhythmias (8,9). However, little is known regarding the effects of heptanol on the arrhythmias induced by ischemia. The aim of this study was to investigate the effects of heptanol on ventricular arrhythmias induced by ischemia and evaluate the changes in connexin 43 (Cx43), the major gap junction protein, in the ischemic myocardium. As heptanol may act on sodium and calcium channels (9–11), it may also affect the action potential. Therefore, the cardiac electrophysiological properties, such as heart rate (HR), PR interval, QT interval and monophasic action potential duration at 90% repolarization (MAPD90) were also assessed.

## Materials and methods

### Animals

A total of 60 adult male Sprague-Dawley (SD) rats, weighing 0.2–0.3 kg, were provided by the Experimental Animal Center of Tongji Hospital (Shanghai, China). All the animal experiments were conducted in compliance with the Guide for the Care and Use of Laboratory Animals (National Reasearch Council, 1996).

### Isolated heart preparation

The SD rats were anesthetized with 1% pentobarbital (0.5 ml/kg) and heparinized via intraperitoneal injection (50 IU/kg). The hearts were quickly excised, mounted on a Langendorff apparatus via the aorta and perfused with Krebs-Henseleit (K-H) buffer (Shanghai Chemical Reagent Co., Shanghai, China) at a constant perfusion pressure of 90 cmH_2_O. The K-H solution consisted of 118.6 mmol/l NaCl, 25 mmol/l NaHCO_3_, 4.7 mmol/l KCl, 1.18 mmol/l KH_2_SO_4_, 1.2 mmol/l MgSO_4_, 2.5 mmol/l CaCl_2_ and 11.1 mmol/l glucose, was gassed with 95% O_2_ and 5% CO_2_ (pH 7.4) and maintained at 37±1°C. Bovine serum albumin (BSA; Sigma-Aldrich, St. Louis, MO, USA) was added to the perfusate at a concentration of 6.013×10^−4^ M (40 mg/l) to improve the stability of the hearts.

All the measurements were performed after an initial stabilization period of at least 15 min of perfusion with stable electrophysiological signals, temperature and coronary flow.

The hearts were randomly divided into 5 groups as follows: i) control group: the hearts were subjected to normal perfusion with K-H buffer solution for 45 min; ii) ischemia group: following perfusion for 15 min with K-H buffer solution, the hearts were subjected to regional ischemia by ligating the left anterior descending coronary artery (LAD) close to its origin for 30 min; iii–v) heptanol groups: the hearts were pretreated with 0.1, 0.3 and 0.5 mM heptanol, respectively, for 15 min prior to the induction of ischemia. Heptanol was dissolved directly in the perfusate at different concentrations and perfused into the isolated hearts.

### Electrophysiological measurements

An epicardial electrogram was recorded using two silver electrodes (with a diameter of 0.3 mm) placed on the surface of the left and right ventricles. The MAPDs were recorded by another two electrodes placed on the surface of the left ventricle near the septal and the aortic cannulae. The epicardial electrograms and MAPD were amplified and analyzed using Medlab computer software (Nanjing Madease Science and Technology Co., Ltd., Nanjing, China).

The parameters, including HR, PR interval, QT interval and MAPD, were recorded at baseline (15 min prior to ischemia; 0 min) and at 10 min (Is10 min), 20 min (Is20 min) and 30 min (Is30 min) after the induction of ischemia. During this period, VT and VF were recorded. VT was defined as a run of ventricular beats lasting >1 min.

### Immunofluorescence analysis

At the end of the experiment, 5 hearts from each group were selected and perfused with 1% Evans blue dye. The non-blue parts were re-stained with triphenyltetrazolium chloride (TTC; Shanghai Chemical Reagent Co.). The sections that stained with TTC were identified as ischemic and were fixed in 10% neutral buffered formalin for immunofluorescence (12,13). A rabbit polyclonal antibody (Zymed Laboratories, San Francisco, CA, USA) directed against Cx43 was used as the primary antibody (dilution, 1:100). FITC-labeled goat anti-rabbit IgG was used as the secondary antibody (dilution, 1:100). Images were captured with a TCS SP2 confocal microscope (Leica, Mannheim, Germany) at a magnification of ×400, using a 40× oil immersion lens. Five areas were analyzed in each heart, for a total of 25 test areas in each group.

### Semi-quantitative reverse transcription-polymerase chain reaction (RT-PCR)

RNA samples (2 μg per experiment) were extracted from the ischemic myocardium. RNA extraction, first-strand complementary DNA (cDNA) synthesis and DNA amplification were performed as previously described, with minor modifications (13). Two pairs of primers designed with Primer 5.0 software were used to amplify a 588-bp product of Cx43 and a 770-bp product of β-actin, which was used as control (Table I). The reaction system (50 μl) contained 33.75 μl H_2_O, 5 μl 10X buffer, 200 μM dNTP mixture, 1.875 mM MgCl_2_, 0.5 μl *Taq* DNA polymerase, 2 μl of the pair primers and 4 μl cDNA. The PCR samples were subjected to initial denaturation for 2 min at 95°C, 30 cycles of 30 sec at 95°C and 30 sec at 60°C, followed by a final extension at 72°C for 5 min. These procedures were completed in a PTC-100 automated thermocycler (MJ Research, Watertown, MA, USA).

The RT-PCR products were visualized on 1.5% agarose gels electrophoresed in 1X Tris-acetate-EDTA buffer. After 25 min, the gels were placed in a solution containing 0.5 μg/ml ethidium bromide and then into a UV transilluminator (Shanghai Qin Xiang Scientific Instrument Co., Ltd., Shanghai, China). The results of the immunofluorescence and RT-PCR analyses were assessed with Leica Qwin image software (Leica Microsystems).

### Statistical analyses

The values are expressed as means ± SE. The myocardial electrical characteristics and Cx43 protein and mRNA expression were compared among groups by the analysis of repeated measures. The occurance of ventricular arrhythmias among groups was assessed by the Fisher’s exact test. P<0.05 was considered to indicate a statistically significant difference.

## Results

### Experimental hearts

The total number of rats used in this experiment was 60. Of the 60 hearts, 8 were discarded due to ligation failure. The hearts that completed the entire protocol (n=52) included 11 hearts in the ischemia group, 10 hearts in the 0.1 mM heptanol group, 10 hearts in the 0.3 mM heptanol group, 9 hearts in the 0.5 mM heptanol group and 12 hearts in the control group without ligation.

### Electrophysiological parameters

**Figure 1:**
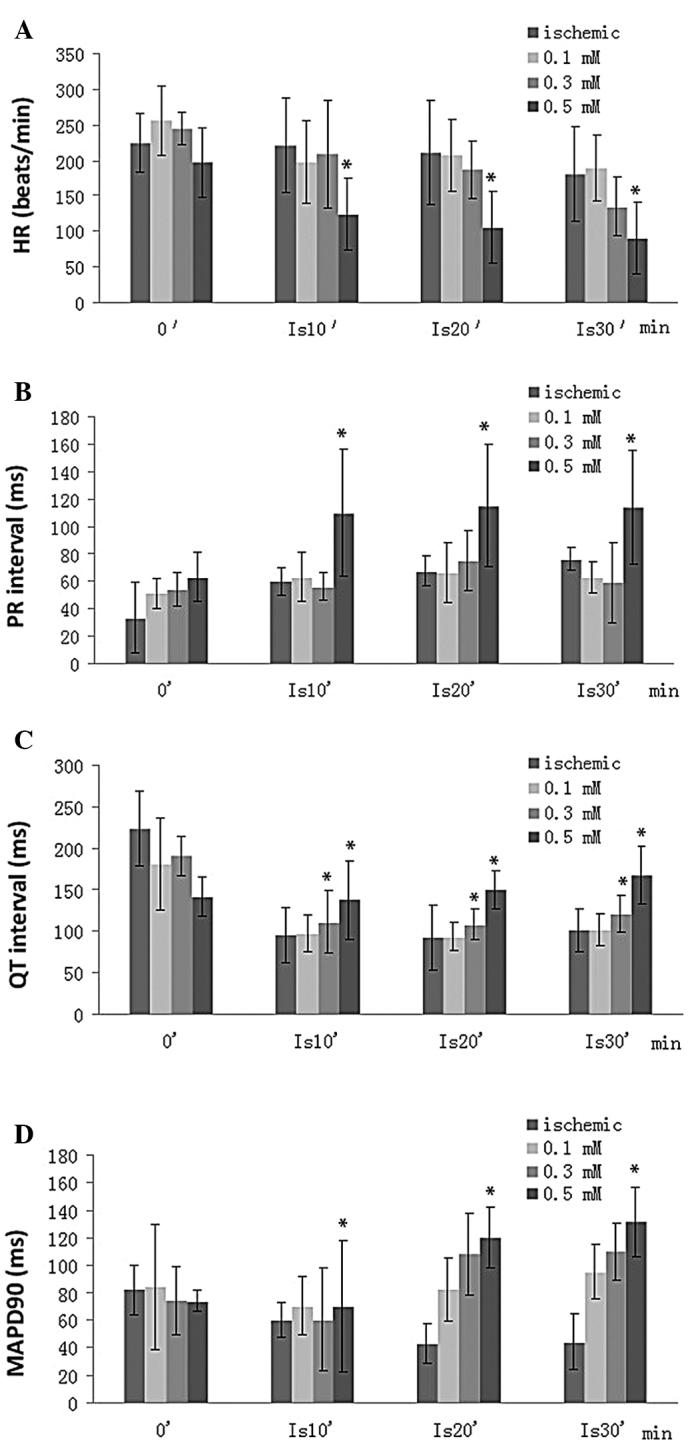
Effect of heptanol on electrophysiological parameters. (A) Effect of heptanol on HR prior to and during 30 min of ischemia. 0′, baseline; Is10′, Is20′ and Is30′, measurement points at 10, 20 and 30 min, respectively, following induction of ischemia through ligation of the left anterior descending coronary artery. (B) Effect of heptanol on PR interval in regional ischemic hearts. (C) Effect of heptanol on QT interval in regional ischemic hearts. (D) Effect of heptanol on MAPD90 in regional ischemic heart. ^*^P<0.05 vs. the ischemic group. HR, heart rate; MAPD90, monophasic action potential duration at 90% repolarization.

The electrophysiological parameters are presented in Table II. Ischemia was shown to decrease the QT interval and MAPD90 and prolong the PR interval; however, it did not affect HR. Heptanol decreased HR following LAD ligation (Fig. 1A), whereas it prolonged the PR interval, QT interval and MAPD90 following LAD ligation, particularly at the concentration of 0.5 mM (Fig. 1B–D).

### Incidence of VT and VF

Heptanol decreased the percentage of ventricular arrhythmias induced by ischemia. The percentage of ventricular arrhythmias was 45% in the ischemia group, 10% in the 0.1 mM group and 0% in the 0.3 and 0.5 mM groups (P<0.05) (Table III).

### Immunofluorescence staining results

**Figure 2:**
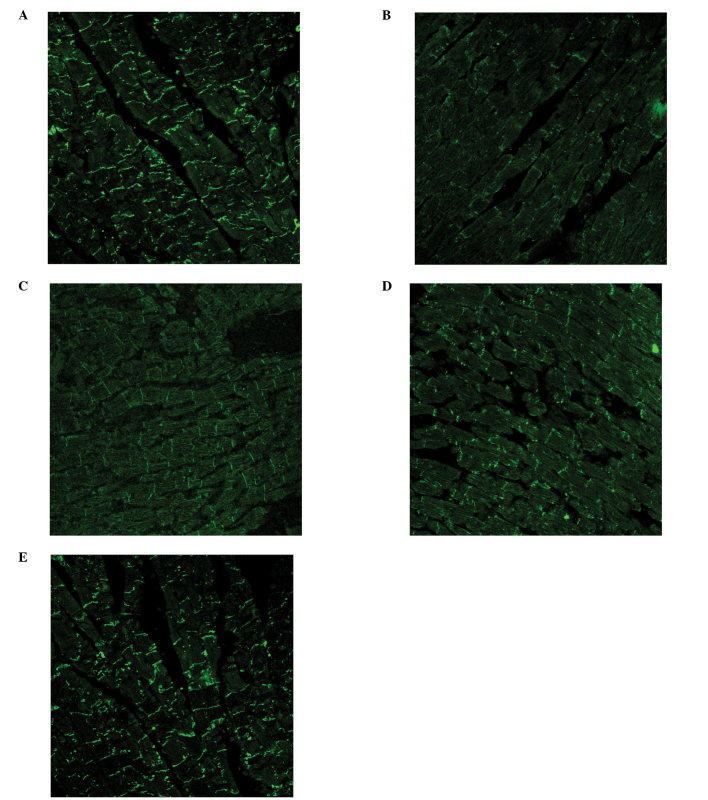
Immunofluorescence image study showing positive staining for connexin 43 (Cx43) protein. (A) Control group: normal myocardium. (B) Ischemic group: Cx43 protein staining was significantly less intense in the ischemic group compared to that in the normal group. (C) 0.1 mM, (D) 0.3 mM and (E) 0.5 mM heptanol groups: immunofluorescence revealed that the amount of Cx43 protein was significantly higher in the heptanol groups compared to that in the ischemic group.

The level of the Cx43 protein, as evaluated by immunofluorescence microscopy, was found to be lower in the ischemic myocardium compared to that in normal myocardium (Fig. 2A and B). Heptanol was able to partly reverse this downregulation induced by ischemia, with the level of the Cx43 protein being 1,706±397 μM^2^ in the control group, 561±147 μM^2^ in the ischemic group, 1,027±215 μM^2^ in the 0.1 mM group, 1,112±301 μM^2^ in the 0.3 mM group and 1,179±425 μM^2^ in the 0.5 mM group (P<0.05). There was no significant difference among the treated groups (Fig. 2C–E).

### mRNA expression of Cx43

**Figure 3:**
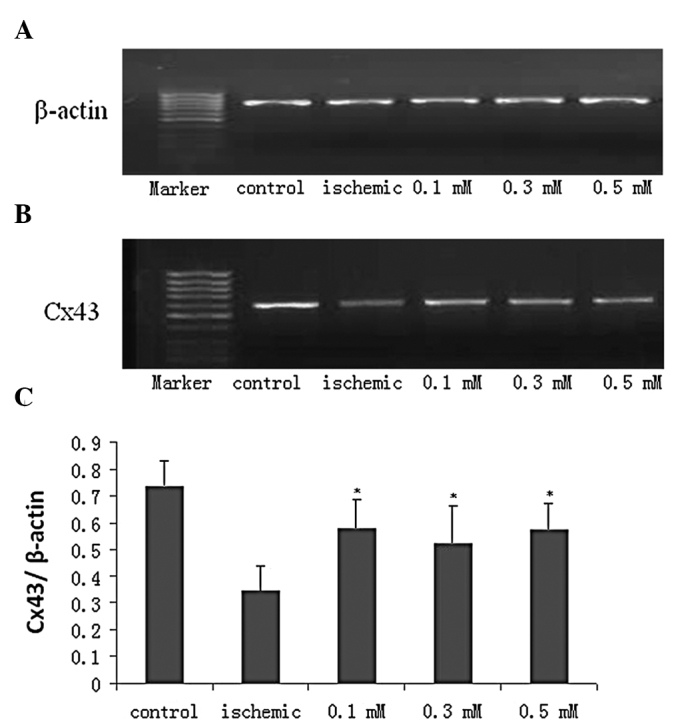
mRNA level of connexin 43 (Cx43) in the ischemic myocardium, with and without heptanol administration. (A) mRNA level of β-actin as control. (B) mRNA level of Cx43. (C) Relative ratio of mRNA level of Cx43 to that of β-actin. The results revealed that ischemia decreased the Cx43 mRNA level and heptanol was able to partly reversed this effect. ^*^P<0.05 vs. the ischemic group.

The results of RT-PCR were expressed by the ratio of Cx43 to β-actin. The mRNA level of Cx43 was found to be lower in the ischemia group compared to that in the control group and heptanol was able to partly reverse this downregulation (Fig. 3A–C).

## Discussion

In this study, we observed that VT and VF occurred in almost half of the cases in the ischemic group and in only one of the 29 cases in the treated groups, suggesting that heptanol significantly decreased the incidence of VT and VF induced by regional ischemia. Previous studies demonstrated that the slower electrical conduction in the ischemic myocardium may lead to reentrant arrhythmia (14,15). Heptanol was shown to reduce the electrical conduction velocity by decreasing the function of gap junctions in all the regions of the myocardium (16). The difference in the reduction of the velocity between normal and ischemic regions in the heart may decrease the incidence of reentrant arrhythmia.

Heptanol was shown to depress the inward Na^+^ current (17), which may explain the prolongation of the QT interval and MAPD90. In the ischemic myocardium, the shorter repolarization duration may lead to the development of reentrant arrhythmia (14). Thus, the decrease in repolarization dispersion between the ischemic and the normal myocardium caused by heptanol may be responsible for the decreased occurrence of ventricular arrhythmia.

Additionally, heptanol reduced the HR and PR interval, suggesting that it may affect the function of the sinus and atrioventricular nodes.

We observed that the expression of Cx43 in the ischemic myocardium was lower compared to that in the normal myocardium, indicating that ischemia may damage gap junctions (18,19). A number of factors may be involved in this change, such as the decrease in the pH and the accumulation of free radicals and lipid metabolites. The reduction of gap junctions in the ischemic myocardium eventually results in a decrease in the conduction velocity. The difference in conduction between the ischemic and the normal myocardium may be an important factor leading to the development of reentrant arrhythmias. In the present experiment, heptanol was able to partly reverse the reduction of Cx43 and may participate in the prevention of ischemic arrhythmias. The result of the RT-PCR revealed that the upregulation of Cx43 by heptanol occurred at the mRNA level. Heptanol is liposoluble; thus, it may cross the cell membrane to regulate the transcription of Cx43.

In conclusion, the gap junction inhibitor heptanol decreases the incidence of VT and VF induced by regional ischemia through altering the myocardial electrophysiological properties and the transcription of Cx43.

## Figures and Tables

**Table I:** Oligonucleotide primers used for RT-PCR analysis.

Target	Primer sequence (5′→3′)	Size (bp)
Cx43	F: TTG TTT CTG TCA CCA GTA AC	588
	R: GAT GAG GAA GGA AGA GAA GC	
β-actin	F: CGT GGC GTT TAC GAA GAT	770
	R: ACC CAG ATC ATG TTT GAG ACC	

RT-PCR, reverse transcription-polymerase chain reaction; Cx43, connexin 43; bp, base pairs.

**Table II:** Electrophysiological parameters in the ischemic group.

		Time points (min)			
		
Parameters	Groups	0	Is10^a^	Is20^b^	Is30^c^
HR	Control	238±54	233±41	218±35	210±53
	Ischemic	225±42	231±51	211±84	181±84
PR interval	Control	36.4±5.2	40.2±5.3	35.9±4.3	41.2±6.3
	Ischemic	33.3±5.9	35.5±8.4	67.1±12.3^d^	76.6±10.3^d^
QT interval	Control	220±48	200±58	198±33	201±57
	Ischemic	224±45	221±68	92±25^d^	101±33^d^
MAPD90	Control	89.3±25.3	88.5±20.3	75.6±20.8	77.6±25.3
	Ischemic	86.7±23.2	78.4±18.9	49.6±18.9^d^	55.2±12.3^d^

a 10 mins after the induction of ischemia.

b 20 mins after the induction of ischemia.

c 30 mins after the induction of ischemia.

d P<0.05 vs. the control group by repeated measures ANOVA.

HR, heart rate; MAPD90, monophasic action potential duration at 90% repolarization.

**Table III:** Incidence of ventricular arrhythmia induced by regional ischemia.

Groups	No.	VT and VF incidence	%
Ischemic	11	5	45
Heptanol			
0.1 mM	10	1	10^a^
0.3 mM	10	0	0^a^
0.5 mM	9	0	0^a^

a P<0.05 vs. the ischemic group by Fisher’s exact test.

VT, ventricular tachycardia; VF, ventricular fibrillation.
